# Special Issue Editorial: Cultural Adaption of Psychological Interventions

**DOI:** 10.32872/cpe.7627

**Published:** 2021-11-23

**Authors:** Eva Heim, Cornelia Weise

**Affiliations:** 1 Institute of Psychology, University of Lausanne, Lausanne, Switzerland; 2 Department of Psychology, University of Zürich, Zürich, Switzerland; 3 Division of Clinical Psychology and Psychotherapy, Department of Psychology, Philipps-University of Marburg, Marburg, Germany

Cultural adaptation of psychological interventions has been discussed controversially in literature. On the one hand, culturally diverse groups are underrepresented in psychological trials, and evidence on acceptability and efficacy of interventions cannot necessarily be transferred from one cultural group to another (Hussain-Gambles et al., 2004; La Roche & Christopher, 2008; Wendler et al., 2006). On the other hand, some researchers are concerned about the fidelity of treatment if culturally adapted (Castro et al., 2010).

There is also considerable debate about what to adapt, and the effect of such adaptations. Empirical evidence on substantial modifications is scarce, and cultural adaptation methods are often insufficiently reported in literature (Chowdhary et al., 2014; Harper Shehadeh et al., 2016). This does not only include adaptations implemented before a trial starts, but also the so called “on-the-fly” adaptations that are done during an ongoing trial.

In this special issue, experiences and empirical evidence on the cultural adaptation of psychological interventions for refugee populations are brought together. In 2016, the German Federal Ministry of Education and Research (FMER) launched a call for research proposals covering the ‘mental health of refugee populations’. Seven research projects were funded. One exclusively focuses on diagnostics, and the other six projects test evidence-based psychological interventions. Each of those six projects consists of three or more sub-projects, which are testing diagnostic tools, the efficacy and cost-effectiveness of interventions, and implementation methods. A total of eleven randomised controlled trials (RCTs) are implemented to test different kinds of psychological interventions among a diversity of target groups, i.e., age groups, specific disorders, or unspecific psychological distress.

In the context of the FMER call, a “task force for cultural adaptions of mental health interventions for refugees” was launched. It pursued two major goals: First, it aimed to develop a common understanding and methodology for documenting and monitoring cultural adaptations in clinical trials. Second, it aimed to integrate the findings of the first step and compile criteria on how cultural adaptations in clinical trials could be reported. The conceptual framework for cultural adaptation by Heim and Kohrt (2019) was used as a basis for this work.

This special issue features experiences, empirical evidence and recommendations resulting from this task force, as well as the final composition of the reporting criteria. The paper by Heim and Knaevelsrud (2021, this issue) provides an introduction to the methodology developed by the task force and describes how cultural adaptations across the different projects and studies were documented and monitored. A content analysis of the documented adaptations is presented in this paper. The subsequent papers highlight five examples of studies that applied the jointly developed cultural adaptation methodology, with different thematic foci. These papers are based on empirical evidence from formative research (e.g., focus groups or key informant interviews) and pilot studies.

Mewes et al. (2021, this issue) describe the development of a culture-sensitive, transdiagnostic intervention to increase knowledge about mental health problems and available treatments. This study highlights the importance of differentiating between the culture-specific adaptation of interventions (for one particular group) and the development of culture-sensitive interventions that can be used for culturally diverse groups.

Kananian et al. (2021, this issue) used Culturally Adapted Cognitive Behavioural Therapy (CA-CBT, Hinton et al., 2012), an intervention that had already been tested among different cultural groups (i.e., Cambodian, Latino, and Arabic-speaking populations). In this study, CA-CBT was prepared to be tested in a new, culturally different group (i.e., Afghan refugees in Germany). Based on a pilot study and focus groups, the intervention was further adapted to be tested in an RCT.

Böttche et al. (2021, this issue) focus on the process from formative research to adaptation. A transdiagnostic intervention, the Common Elements Treatment Approach (CETA, Murray et al., 2014), was adapted for Arabic-speaking refugees in Germany and will be provided both face-to-face and through the internet in a non-inferiority trial. In preparation of this study, cultural concepts of distress were assessed among the target population. The main focus of the paper is on the decision-making process, and the authors provide a summary of their most salient decisions.

The process of adapting an already culturally-sensitive, transdiagnostic treatment to also include substance use disorders is described in the paper by Lotzin et al. (2021, this issue). The authors used Skills-Training of Affect Regulation – A Culture-sensitive Approach (STARC, Koch & Liedl, 2019) in their study. Focus group discussions were conducted to examine culture-specific assumptions about substance use. This data was used to adapt the treatment manual that will be tested in an upcoming RCT.

Unterhitzenberger et al. (2021, this issue) tested Trauma-Focused Cognitive Behavioral Therapy (TF-CBT, Cohen et al., 2017) in a pilot study with unaccompanied refugee minors (URMs). The paper highlights “on-the-fly” adaptations implemented by the therapists during the pilot study. The main adaptation concerned the so-called “crisis of the week”, i.e., participants struggles and concerns in their daily lives. This shows that post-migration stressors are a very important factor when adapting psychological interventions to refugee populations – an aspect that may sometimes be even more relevant than ethnic origin.

Finally, Heim et al. (2021, this issue) present the *Reporting Cultural Adaptation in Psychological Trials* (RECAPT) criteria. The RECAPT criteria were developed jointly by the above-described task force. To achieve a broader consent on the RECAPT criteria, an online survey was conducted among eleven international experts in the field of global mental health and psychological interventions for refugee populations.

In summary, this special issue features the experience of a variety of studies in which a diversity of psychological interventions were culturally adapted and tested among refugee populations in Germany. The task force provided a unique opportunity for exchange and discussions, with the aim of advancing the emerging field of cultural adaptations in mental health interventions. The RECAPT criteria (Heim et al., 2021, this issue) will hopefully contribute to a more systematised and transparent documentation of cultural adaptation in psychological research in the future. This is an important precondition to enhance the empirical evidence concerning the effect of such adaptations on the efficacy and acceptability of psychotherapy among culturally diverse groups.
